# Does Internet Use Affect Individuals’ Medical Service Satisfaction? Evidence from China

**DOI:** 10.3390/healthcare8020081

**Published:** 2020-03-31

**Authors:** Hu Liu, Xiaomei Gong, Jiaping Zhang

**Affiliations:** 1 International Business School, Shaanxi Normal University, Xi’an 710119, Shaanxi, China; liuhu@snnu.edu.cn; 2 School of Public Economics and Administration, Shanghai University of Finance and Economics, Shanghai 200433, China; 15191561635@163.com; 3 School of Economics and Management, Tongji University, Shanghai 200092, China; 4 School of Management and Labor Relations, Rutgers University, New Brunswick, NJ 08901, USA

**Keywords:** Internet use, medical service satisfaction, satisfaction, negativity bias, medical risk management

## Abstract

Empirical evidence that combines traditional factors and information technology factors to predict public attitudes toward to medical services is inadequate. To fill this gap, this study investigates the impact of Internet use on people’s satisfaction with medical services by employing the Chinese Social Survey for 2013, 2015 and 2017 (including 28,239 samples in total). Estimation results under the ordered probit reveal that Internet use is negatively correlated with individuals’ medical services satisfaction. The results support the negativity bias theory, namely, compared with positive information, netizens pay more attention to negative medical-related information on the Internet. The results are still reliable by adopting substitution variable methods, subdividing the samples, employing other estimation methods and carrying out placebo tests to conduct robustness checks. This study further enriches the literature on public attitudes toward medical services and provides additional policy implications for medical risk management in the digital era.

## 1. Introduction

The Internet has been closely intertwined with human health activities. Since the end of 2019, the world began to experience a public health emergency caused by a novel coronavirus pneumonia (COVID-19) [1]. In this battle against the infectious disease, the Internet, especially social media, has played an unprecedented role. For example, in China, the Sina MicroBlog (Sina, Beijing, China), a social media, has become an important channel for people to learn about the latest progress of that infectious diseases, spread disease-related knowledge and seek social assistance. At the same time, online public opinion is also affecting China’s health policies and government decisions. However, various rumors and negative information related to 2019-nCoV also appeared on the Internet. For example, there was a rumor that eating radix isatidis and fumigating vinegar can prevent the 2019-ncov [2]. Those rumors and negative information had attracted a large number of netizens’ attention and had aggravated people’s panic about the virus, such as causing people to stock up on radix isatidis [3].

Indeed, the past decade has witnessed the widespread application of the Internet in public health, which has aroused scholars’ persistent interest in exploring the relationship between the Internet and health. A growing number of studies have shown that Internet is widely utilized by its users to search for the information related to drugs and health [4,5,6,7,8,9,10]. Meanwhile, various kinds of Internet apps have also become important instruments for people to engage in health management in their daily life (e.g., telemedicine and electronic health records) [11]. However, excessive use of the Internet or Internet addiction may also have a negative impact on users’ mental health (e.g., anxiety and depression) [12,13,14,15,16].

Evidence has shown that people’s evaluation to medical service system is of great significance to both medical risk management and the improvement of medical service situation [17,18,19,20]. However, in terms of empirical research on the impact of Internet use on public health, a neglected point is that Internet use can also have an impact on users’ evaluation or satisfaction with medical service. The “negativity bias” in the social transmission of information and psychology, is a well-known phenomenon, which emphasizes that audiences pay more attention to negative information (or negative stimulate) than positive or neutral information (or positive stimulate), such as official corruption, entertainment gossip and environmental pollution [21,22,23,24,25,26,27]. The prior studies suggested that negative information often represents a dangerous signal and negativity bias is a manifestation of people’s adaptation to the environment, which can trigger people’s instinctive response to biological evolution [21,28,29]. Other scholars including Walker and Blaine [30], Hamlin et al. [31], Fessler et al. [32], Carstensen and Deliema [33] all confirmed the existence of negativity bias effect. Therefore, according to the theory of negativity bias, when netizens search for medical information or manage health through the Internet, they may have more preference for negative information related to medical service (e.g., doctor–patient conflict and outbreak of infectious diseases) inadvertently, thus reducing their satisfaction with the medical service.

Compared with positive stimulus, negative stimulus may cause a stronger emotional response for the audience [34,35], which may lead to the deviation of people’s subjective judgment on things. In addition, when people are in a negative cognitive environment, people’s predictions for the probability of negative or dangerous events will be higher [36,37,38]. Negativity bias is also strongly associated with behaviors such as depression and anxiety [39,40]. For example, a recent study by Zhang et al. [41] shows that Internet use is negatively correlated with Chinese people’ satisfaction with the government’s environmental protection. They hold that netizens pay more attention to news about environmental pollution or government’s negative environmental protection behavior on the Internet.

Therefore, as discussed above, negativity bias effects may indicate a negative correlation between Internet use and individuals’ satisfaction with medical services. However, the existing literature does not directly (to the best of the authors’ knowledge) address the relationship between Internet use and individuals’ satisfaction with medical services. To fill this gap, this study attempts to investigate whether the relationship between Internet use and individuals’ satisfaction with medical service is affected by negativity bias effect in China.

Compared with the existing literature, the contribution of this paper is threefold. First, this paper provides new evidence for the negativity bias theory. Second, this study further enriches the literature on medical risk, indicating that medical risk management in the digital era needs to take both traditional factors and Internet technology factors into consideration. Third, this paper can provide additional policy implications for medical risk management in the digital era.

## 2. Background: Medical Services and Internet Development Situation in China

During the past 40 years, China has experienced amazing economic achievements, which has greatly improved people’s income and living standard. According to the general law of economic development, with the improvement of people’s economic situation, people’s demand for health-related services will increase [42]. Nevertheless, the current medical services quality in China has not kept pace with its economic development speed [43]. Chinese society is still facing the contradiction between people’s increasing demand for medical services and the shortage of medical resources supply [44,45]. As a result, the difficulty and expensiveness of getting medical service have deeply troubled Chinese residents in the past few years. Moreover, since China has not yet established a perfect mechanism to resolve medical disputes and prevent the medical corruption, both the tense doctor–patient relationship and drug safety have become serious problems for Chinese society in recent years [46]. Perhaps because of people’s personal experience about the current medical services situation, every news report on medical disputes and drug safety often attracts extensive attention and discussion in Chinese society, which has accelerated the formulation and improvement of China’s medical policies. For example, in 2018, the disclosure of “Changchun Changsheng vaccine incident”, an incident of fake records of rabies vaccine production in China, caused a great uproar in the society, which was also listed as the top ten buzzwords of social life of the year [47].

Meanwhile, China has also witnessed the rapid development and application of Internet technology during the past ten years [48]. As of 2018, the number of Chinese Internet users had reached 828.51 million [49], making China the world’s largest Internet user. In particular, the current Chinese government attaches a great importance to promoting the construction of information (e.g., the “Internet +”, “digital China” and “sharing economy”), aiming at realizing the all-round development of Chinese society and the improvement of people’s living standards through Internet technology. In the field of medical, Chinese government is also vigorously promoting the development of “Internet + medical” to improve the quality and coverage of medical services in China. In addition, telemedicine has also been widely developed in China and data showed that the market size of China’s Internet medical exceeded 30 billion yuan in 2017 [50].

## 3. Methodology

### 3.1. Data Source

Data applied in this study is derived from the Chinese Social Survey (CSS) [51], which is a nationwide large-scale continuous sampling survey project in China (http://css.cssn.cn/css_sy/). The multi-stage compound sampling method is utilized in CSS and respondents are Chinese citizens aged 18 and above. This survey covers a wide range of information about respondents, including family, occupation, income, consumption and attitudes, etc. Nowadays, CSS has become an important data source for the study of Chinese social development, e.g., Zhang et al. [52].

So far, CSS has released the latest data to 2017. However, only CSS for 2013, 2015 and 2017 included respondents’ information related to both Internet use and assessment on medical service. Therefore, in this study, we use data from the survey conducted in 2013, 2015 and 2017 (i.e., CSS2013, CSS2015 and CSS2017). The CSS2013 had interviewed 10,206 samples from 30 provinces in mainland China (excluding Xinjiang, Hong Kong, Macao and Taiwan), ranging in age from 18 to 72 years, including 4565 male samples and 5641 female samples. A total of 10,243 samples were included in CSS2015, ranging in age from 19 to 70, including 4651 male samples and 5592 female samples. While a total of 10,143 samples were interviewed in CSS2017, including 4536 male samples and 5607 female samples.

### 3.2. Variable Setting

#### 3.2.1. Dependent Variable

The dependent variable, medical service satisfaction, was obtained by respondents’ subjective evaluation with medical service (denoted by MSS). The responses ranged from “very dissatisfied” = 1 to “very satisfied” = 10, namely the higher the value of the dependent variable, the higher the respondents’ satisfaction with medical service.

#### 3.2.2. Explanatory Variable

Referencing to Tranos and Stich [53], the key explanatory variable, Internet use, is set as a dummy variable, namely, it is equal to 1 when the respondent uses the Internet, otherwise 0 (denoted by IU). The control variables mainly include the following several factors: (1) individual characteristics, including gender, age, education, political identity, household registration; (2) socio-economic factors, including family economic level, whether to buy medical insurance or not. In addition, this study also controls several province dummy variables and year dummy variables. In this paper, samples with uncertain answers and missing values were excluded. Finally, 28,239 samples are obtained, including 10,427 netizens and 17,812 non-netizens for the baseline regression.

### 3.3. Model Introduction and Estimation Method

Considering that the dependent variable is an ordered variable from 1 to 10, consistent with most prior studies (e.g., Asadullah et al. [54]), this study also adopts the ordered probit to estimate the effect of Internet use on medical service satisfaction. Specifically, this study constructs the following econometric model:

(1) $$MSS^{*}{}_{i}=\beta_{0}+\beta_{1}IU_{i}+\lambda X+\varepsilon_{i},$$

where *i* represents the *ith* respondent, and MSS^*^ is the latent variable of *MSS*. The variable of main interest is *IU* and *β*_1_ is the key parameter concerned in this study. When *β*_1_ is significantly less than 0, it indicates that there is a negative correlation between Internet use and medical service satisfaction. A series of control variables that may affect people’s satisfaction with medical service are included in vector X, and the $\varepsilon_{i}$ indexes the error term. In addition, MSS^*^ and MSS satisfy the following relationship:

(2) $$MSS_{i}=\{\begin{array}{cc} =1, & \;{MSS}_{i}^{*}\leq R_{1}\\ =2, & R_{1}<{MSS}_{i}^{*}\leq R_{2}\\ & \ldots \ldots \\ =9, & R_{8}<{MSS}_{i}^{*}\leq R_{9}\\ =10, & \;R_{9}<{MSS}_{i}^{*} \end{array},$$

where *R_1_*–*R_9_* are the parameters (cutoff points) that need to be estimated and satisfy *R_1_<R_2_<……<R_8_<R_9_*.

### 3.4. Methods for the Robustness Check

In order to verify the reliability of the estimation results, several robustness checks are also conducted in this study. Generally speaking, methods used in previous studies for robustness checks mainly include: Substitution variable method, subdividing the samples and adopting different estimation methods, e.g., Zhang et al [52], Zheng et al. [55]. Therefore, this paper mainly uses the following methods to conduct the robustness checks:

First, this study employs other variables to replace the Internet use. Generally speaking, if netizens’ satisfaction with medical service is lower mainly because they are influenced by negative news or information on the Internet, would residents who have doubts about network information be more satisfied with medical service? If this logic is verified, the existence of negativity bias effect among Chinese netizens can be further demonstrated. According to CSS, by asking the respondents whether they agree that Internet information is not as reliable as that of traditional media (e.g., TV, radio, newspapers) and whether they agree with the opinions of Internet users cannot represent all the people, this paper constructs respondents’ two attitudes toward the Internet information (denoted by attitudes toward Internet information and attitudes toward netizens) to replace the key explanatory variable. Both of the two variables are categorical indicators with values ranging from 1 to 4 (strongly disagree; disagree; agree; strongly agree). The higher the value, the less likely respondents are to trust the Internet information.

Second, this paper also adopts other variables to replace the dependent variable. Medical service quality is a comprehensive indicator, which includes not only drug safety and drug price, but also includes service quality of medical institutions, doctors’ medical ethics and professional skills level, etc. Therefore, this paper further uses respondents’ evaluation on different aspects of medical service to replace the dependent variable. Specifically, according to CSS, five categories of subdivided dimensions are employed to further reflect medical service, including service quality of medical institutions, convenience of seeking medical service, medical safety, doctor–patient relationship and the supply level of government medical services. The definitions of the main variables are shown in Table 1.

Third, considering that previous studies have shown that negativity bias varies among different populations [52], this study further subdivides the samples according to gender, urban-rural and education to conduct the robustness checks. In addition, we also estimate the impact of Internet use on medical service satisfaction in different years for robustness check.

Fourth, in the benchmark model, we use ordered probit for estimation; this paper further uses the ordered logit to conduct the robustness check. In addition, medical service satisfaction is set as a dummy variable (MSS1), namely, when the medical service satisfaction is greater than 5 (i.e., high medical service satisfaction), it is 1; otherwise (i.e., low medical service satisfaction), it is 0. Further, we employ the probit model to investigate whether Internet use reduce the probability of individuals’ high satisfaction with medical services.

Fifth, considering that people’s Internet use may be the result of “self-selection”, for example, residents with higher family economic level and education level have greater opportunities to use the Internet. As a result, endogenous problems can arise because of the selective bias of samples. Therefore, this paper further uses the propensity score matching (PSM) method to solve the problem of selective bias [56,57].

Sixth, as discussed above, if the negative impact of Internet use on medical service satisfaction is due to negativity bias, what will be the impact of traditional media use on medical service satisfaction? Generally speaking, traditional media are often under the strict control of the authorities and mainly focus on reporting the positive information. Therefore, this paper further investigates the impact of traditional media use on individuals’ medical service satisfaction to conduct a placebo test. If the traditional media use has a positive effect on medical services satisfaction or the effect is not significant, then the conclusion that Internet use has a negative effect on the medical services satisfaction can be further verified from an opposite perspective. According to the CSS, four variables are adopted in this paper to reflect the frequency of interviewees watching TV, listening to the radio, reading newspapers (magazines) and reading books (denoted by watching TV, listening radio, reading newspapers and reading books, respectively). Each of the above variables is an ordered variable from 1 to 6, namely, from 1 = never to 6 = every day.

## 4. Results

### 4.1. Statistical Analyses

The descriptive statistical of main variables are shown in Table 2. It can be found that the average medical service satisfaction of the sample was 6.40, which is higher than the median (*MSS* = 5). The average of the five variables reflecting the satisfaction with the service quality of medical institutions are all higher than 5; the other eight variables reflecting satisfaction with the convenience of seeking medical service, medical safety, doctor–patient relationship and the supply level of government medical services are all higher than 2. The proportion of Internet users in the samples was 37%; 48% of the samples belong to urban residents.

Further, netizens and non-netizens have different statistical values for each variable. The average medical services satisfaction of non-netizens is 6.55, which is higher than that of netizens (6.14). Analogously, except for X5 and X7, non-netizens are also more satisfied with different types of medical services than netizens. Overall, netizens are younger and more educated than non-netizens. In addition, the proportion of urban residents and party members among netizens is higher than that of non-netizens.

**Figure 1:**
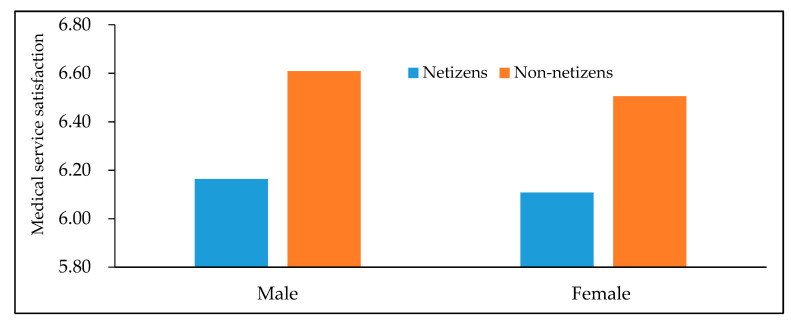
Differences in medical service satisfaction between netizens and non-netizens (by gender). Data are from the Chinese Social Survey [51] for 2013, 2015 and 2017.

**Figure 2:**
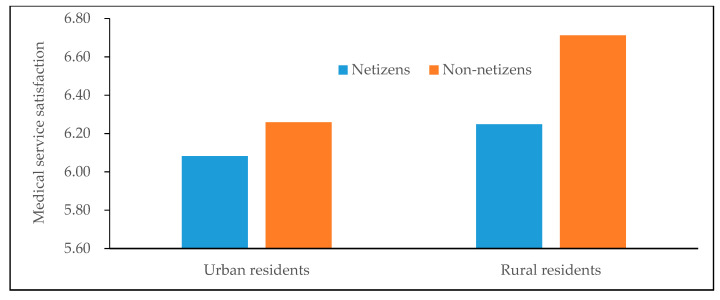
Differences in medical service satisfaction between netizens and non-netizens (by urban-rural). Data are from the Chinese Social Survey [51] for 2013, 2015 and 2017.

**Figure 3:**
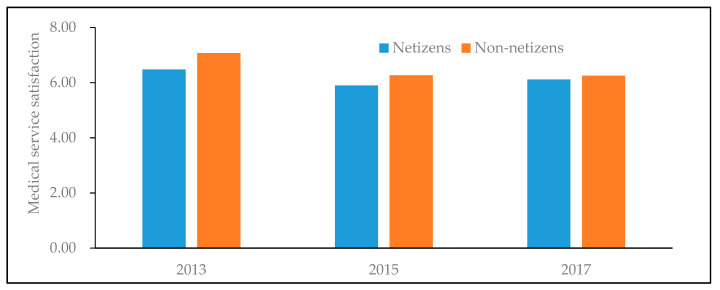
Differences in medical service satisfaction between netizens and non-netizens in different years. Data are from the Chinese Social Survey [51] for 2013, 2015 and 2017.

Figure 1 and Figure 2 depict the difference of average medical service satisfaction between netizens and non-netizens. It can be found that for men, women, urban residents and rural residents, the average medical service satisfaction of non-netizens is all higher than that of netizens, namely there is a negative statistical relationship between Internet use and medical service satisfaction. Analogously, Figure 3 also shows that in different years, the medical service satisfaction of Internet users is also lower than that of non-Internet users.

### 4.2. Baseline Regression Results

The estimation results of the benchmark model are shown in Table 3. Column (1) only examines the impact of Internet use on medical service satisfaction and column (2)–(4) adds control variables in turn. It can be found that the key explanatory variable, Internet use, is significantly negative in columns (1)–(4), indicating that the estimation results are relatively robust. From the results in the columns (4), the influence coefficient of Internet use on medical security satisfaction is –0.1364 (significant at the 1%). The results imply that compared with non-Internet users, the medical service satisfaction of Internet users is 0.1364 lower, which is consistent with the descriptive statistics above. Therefore, the empirical results of the benchmark model support the theory of negativity bias, indicating that Chinese netizens would be affected by negative news related to medical service, such as medical disputes and expensive medical treatment.

For the results of control variables, both educated and urban residents are less satisfied with medical service than illiterate and rural residents, respectively. However, the satisfaction of people who are party members, have high income and have medical insurance to medical service would be higher.

### 4.3. Robustness Checks

#### 4.3.1. Substitution Variable Method Ⅰ

As discussed above, the estimation results of the impact of attitudes toward Internet on medical service satisfaction are shown in Table 4. As shown in Table 4, the coefficients of the two variables reflecting respondents’ attitudes toward Internet information are all significantly positive. It indicates that when people are more suspicious about the Internet information, they are more satisfied with medical service. Therefore, the results in Table 4 are consistent with the above conclusions, indicating that Chinese netizens’ evaluation of medical service would be affected by negativity bias.

#### 4.3.2. Substitution Variable Method Ⅱ

The estimation results of the impact of Internet use on people’s different aspects of medical service satisfaction are reported in Table 5. First, results in columns (1)–(6) show that, except for the indicator of “evaluation on hospital equipment”, the impact coefficients of Internet use on other five indicators reflecting the service quality of medical institutions are all significantly negative. For the influence of the Internet on the four indicators reflecting the convenience of seeking medical service, except for the “distance from hospital”, the other coefficients are all significantly negative. Finally, the Internet use has a significant negative impact on people’s evaluation to medical security, doctor–patient relationship and the supply level of government medical services. In general, the above analysis indicates that netizens are less satisfied with the five categories of indicators reflecting medical service, which further supports the negativity bias theory.

#### 4.3.3. Subdivided Sample Analysis.

Columns (1)–(4) in Table 6 report the estimation results of Internet use on medical service satisfaction for male, female, urban and rural samples, while columns (5)–(7) are the estimation results for samples in 2013, 2015 and 2017, respectively. The coefficients of Internet use are all significantly negative in columns (1)–(7) of Table 6. Table 7 shows the impact of Internet use on medical service satisfaction to different educated populations. The coefficients of Internet us are all negative in columns (1)–(3) of Table 7, but it is not significant for people with an education level of undergraduates and above. The results indicate that the negative impact of Internet use on medical service satisfaction among low-educated people is more obvious.

#### 4.3.4. Internet Use and Medical Service Satisfaction (Ordered Logit and Probit Estimation)

As shown in Table 8, consistent with the baseline model above, columns (1) and (2) indicate that there is a significant negative relationship between Internet use and medical service satisfaction under ordered logit estimation. In addition, the coefficients of Internet use in columns (3) and (4) are all negative and significant at 1%. The results further suggest that compared with Internet users, non-Internet users are more likely to have high satisfaction with medical services.

#### 4.3.5. PSM Analysis.

Table 9 shows the estimation results under the PSM. As a comparison, this study simultaneously reports the estimation results by four matching methods, including 2-nearest neighbor matching, radius matching, kernel matching and local linear regression matching. Above all, to ensure the validity of propensity score matching, the balance of related control variables needs to be tested. The results show that there are significant differences in all covariates before matching. The proportion of standardized bias (%bias) of all covariates (except for medical insurance) are higher than 10% and the t-test results of all variables reject the null hypothesis that there is no systematic difference between the treatment group and the control group. However, the standardized bias of all explanatory variables after matching are less than 10% and most t test results do not reject that there is no systematic difference between the treatment group and the control group. The results show that the average treatment effects on the treated (ATT) under the four matching methods are significantly negative, with the coefficients ranging from –0.3772 to –0.3332. Therefore, after eliminating the systematic differences among the samples, compared with non-netizens, netizens still have lower satisfaction with medical service.

#### 4.3.6. Internet Use and Medical Service Satisfaction: Placebo Test

As shown in Table 10, the coefficients of the four variables reflecting the frequency of traditional media use are all positive. Moreover, both watching TV and reading books have a significant positive effect on medical services satisfaction. Therefore, the placebo test further implies that the negative correlation between Internet use and medical service satisfaction is robust.

## 5. Discussion

Public evaluation on medical services is of great significance to health management in a region or country. However, previous studies have lacked research that considers the impact of information technology factors on public attitudes to medical services. Based on CSS for 2013, 2015 and 2017, This paper adopts econometric methods to investigate the impact of Internet use on individuals’ medical services satisfaction. Our study suggests a negative correlation between Internet use and individuals’ satisfaction with medical services. The results are consistent with the negativity bias theory, namely, compared with positive and neutral information, people will pay more attention to negative information related to medical services on the Internet. As a result, Internet use may aggravate individuals’ dissatisfaction with the current medical services and several challenges to public risk management.

This study is in line with several prior researches. First, the results are consistent with the previous studies on negativity bias in Chinese context. For example, Zhang et al. [41] found that netizens have a lower evaluation of the government’s environmental protection in China. Zhang et [52] suggested a negative correlation between Internet use and public food safety perception in China. Second, the results further support several previous studies on the impact of media exposure, especially social media, on public risk perception. For example, Choi et al. [58] found that social media can significantly enhance people’s risk perception during Middle East respiratory syndrome (MERS) outbreak in South Korean. Others studies such as Christensen et al. [59], Bago and Lompo [60], Chen and Stoecker [61], all found media exposure can increase the public risk perception related health information. Third, a great number of studies have found that Internet use is associated with negative outcomes (e.g., depression) of individuals’ mental health, such as Seki et al. [62], Yücens and Üzer [63]. Therefore, this study can provide additional evidence for how Internet use affects individuals’ mental health. Fourth, this paper also finds that the impact of Internet use on medical service satisfaction among people with different levels of education varies, namely, the negative impact of Internet use on medical service satisfaction among low-educated people is more obvious. The result is in line with Zhang et al. [52]. This can be explained by the fact that for low-educated populations, they may lack rational judgments when facing negative information or rumors on the Internet.

In the digital era, the Internet is not only an important driving force for economic development, but also may have an important impact on people’s values or attitudes to participate in public governance, such as the satisfaction with medical services in this study. This article can provide several implications for future public health management. On the one hand, public risk management should consider the impact of technical factors such as the Internet. The public sector can improve health service through e-government, promptly respond to hot topics of public concern and reduce the information asymmetry among society sectors. On the other hand, the public sector should moderately guide netizens to rationally view Internet information in the digital era. For example, investment in education should be constantly strengthened. In general, the more educated people are, the more rational they are likely to be in the face of negative news. In addition, the popularization of health knowledge to the public can also contribute to citizens’ objective view of negative medical information on the Internet.

## 6. Limitations and Future Research

Several limitations still exist in this study. First, although this paper finds a significant negative correlation between Internet use and medical service satisfaction, the values of regression coefficient is relatively low. Second, this study adopts cross-section data to investigate the relationship between Internet use and medical service satisfaction. Therefore, this paper may only find correlation, not causality. Future research may use panel data to further identify the causal relationship between Internet use and medical service satisfaction. Third, due to data limitations, the core explanatory variable of this article is only set up as a dummy variable, which is difficult to examine the impact of online time length and online content on personal medical service satisfaction. Future research can carry out more targeted investigations to further study this topic. In addition, future research investigating the impact of specific social media use, such as Sina MicroBlog (Sina, Beijing, China), on personal medical service satisfaction is also encouraged. Finally, future research on this topic targeting specific populations is also of great significance. For example, many countries around the world are experiencing a public health crisis caused by COVID-19. Therefore, study that specifically investigates the relationship between negativity bias and mental health for populations infected with COVID-19 can also contribute to public health management.

## 7. Conclusions

This study suggests a significant negative correlation between Internet use and individuals’ medical services satisfaction. The results support the negativity bias theory, indicating that compared with positive information, netizens pay more attention to negative medical-related information on the Internet. The results are still reliable by adopting substitution variable method, subdividing the samples, employing other estimation methods and carrying out placebo test to conduct the robustness checks.

## Figures and Tables

**Table 1:** Definition of variables.

Variable	Definition
MSS	Ten categories: From very dissatisfied = 1 to very satisfied = 10.
IU	Using the Internet = 1, else = 0
Gender	Male = 1, female = 0
Age	Respondents’ age
Education	Six categories: Illiteracy = 1, primary school = 2, junior high school = 3, senior high school=4, undergraduate = 5, graduate = 6
Political identity	Party member = 1, else = 0
Household registration	Urban = 1, rural = 0
Family economic level	Five categories: Low = 1 and high = 5.
Medical insurance	Have medical insurance = 1, else = 0
Attitudes toward Internet information	Four categories: Strongly disagree = 1 and strongly agree = 4.
Attitudes toward netizens	Four categories: Strongly disagree = 1 and strongly agree = 4.
Watching TV	Six categories: Never= 1 and every day = 6.
Listening radio,	Six categories: Never= 1 and every day = 6.
Reading newspapers	Six categories: Never= 1 and every day = 6.
Reading books	Six categories: Never= 1 and every day = 6.
**Service quality of medical institutions (when respondents went to a medical institution recently)**	
Doctor attitude (X1)	Evaluation on the attitude of medical staff. Ten categories: 1 = “very dissatisfied” to 10 = “very satisfied”
Doctors’ professional skills (X2)	Evaluation of doctor’s professional skills. Ten categories: 1 = “very dissatisfied” to 10= “very satisfied”
Doctors’ medical ethics (X3)	Evaluation of doctor’s medical ethics. Ten categories: 1 = “very dissatisfied” to 10= “very satisfied”
Hospital environment (X4)	Evaluation of hospital environment. Ten categories: 1 = “very dissatisfied” to 10= “very satisfied”
Hospital equipment (X5)	Evaluation of hospital equipment. Ten categories: 1 = “very dissatisfied” to 10= “very satisfied”
Order of medical treatment (X6)	Evaluation of the order of medical treatment Ten categories: 1 = “very dissatisfied” to 10= “very satisfied”
**Convenience of seeking medical service**	
Distance from hospital (X7)	Do you agree that it is too far from the clinic? Four categories: 1 = “very agree” to 4 = “very disagree”
Appointment time (X8)	Do you agree that the appointment time is too long? Four categories: 1 = “very agree” to 4 = “very disagree”.
Waiting time (X9)	Do you agree that the waiting time is too long? Four categories: 1 = “very agree” to 4 = “very disagree”
Medical expense (x10)	Do you agree medical expenses are too expensive Four categories: 1 = “very agree” to 4 = “very disagree”
**Medical safety** (X11)	Evaluation of medical safety. Four categories: 1 = “very unsafe” to 4= “very safe”
**Doctor-patient relationship** (X12)	Degree of trust in doctors. Four categories: 1 = “very distrust” to 4= “very trust”
**Supply level of government** **medical services** (X13)	Evaluation of the work for local government in providing medical services. Four categories: 1= “very bad” to 4= “very good”.

Data are from the Chinese Social Survey [51] for 2013, 2015 and 2017.

**Table 2:** Descriptive statistics of the main variables.

Variable	Total Sample		Netizens		Non-netizens	
	Mean	SD	Mean	SD	Mean	SD
MSS	6.40	2.47	6.14	2.35	6.55	2.52
IU	0.37	0.48				
Gender	0.45	0.50	0.49	0.50	0.43	0.50
Age	46.27	13.80	36.21	12.03	52.16	11.10
Education	2.99	1.22	3.92	1.02	2.46	0.97
Political identity	0.10	0.30	0.15	0.36	0.07	0.25
Household registration	0.48	0.50	0.67	0.47	0.36	0.48
Family economic level	2.18	0.91	2.35	0.86	2.09	0.92
Medical insurance	0.88	0.32	0.87	0.34	0.89	0.31
X1	7.10	2.06	6.74	2.12	7.26	2.01
X2	6.93	1.92	6.72	1.93	7.02	1.91
X3	7.04	2.06	6.69	2.17	7.20	1.99
X4	7.04	1.89	6.83	1.91	7.14	1.87
X5	6.84	1.96	6.84	1.89	6.84	1.99
X6	7.07	1.95	6.73	2.07	7.22	1.87
X7	3.08	0.87	3.17	0.81	3.04	0.90
X8	3.18	0.86	3.04	0.93	3.24	0.82
X9	2.99	0.93	2.78	0.99	3.09	0.89
X10	2.29	0.95	2.24	0.94	2.32	0.96
X11	2.87	0.59	2.72	0.59	2.93	0.58
X12	3.01	0.69	2.87	0.66	3.09	0.69
X13	2.80	0.67	2.71	0.63	2.85	0.66
Attitudes toward Internet information	2.70	0.76	2.70	0.76	2.27	0.70
Attitudes toward netizens	2.99	0.77	2.99	0.77	2.53	0.74
Watching TV	5.40	1.21	5.21	1.35	5.49	1.13
Listening radio,	1.55	1.40	1.74	1.52	1.46	1.33
Reading newspapers	2.25	1.82	3.40	1.92	1.73	1.51
Reading books	2.19	1.76	3.47	1.91	1.61	1.33

Data are from the Chinese Social Survey [51] for 2013, 2015 and 2017. SD—standard deviation. MSS—medical service satisfaction. IU—Internet use.

**Table 3:** Internet use and medical service satisfaction (ordered probit).

Variable	Dependent variable: MSS			
	(1)	(2)	(3)	(4)
IU	–0.1503^***^	–0.1011^***^	–0.1306^***^	–0.1364^***^
	(0.0126)	(0.0173)	(0.0174)	(0.0174)
Gender		0.0313^**^	0.0471^***^	0.0489^***^
		(0.0128)	(0.0128)	(0.0128)
Age (reference for younger than 31 years old)				
30 < Age < 45		–0.1070^***^	–0.1116^***^	–0.1315^***^
		(0.0192)	(0.0192)	(0.0192)
44 < Age < 65		–0.0278	–0.0390^*^	–0.0700^***^
		(0.0200)	(0.0200)	(0.0202)
64 < Age		0.0681^**^	0.0435	0.0088
		(0.0289)	(0.0290)	(0.0291)
Education (reference for the illiteracy)				
Primary school		–0.0531^**^	–0.0683^***^	–0.0713^***^
		(0.0240)	(0.0241)	(0.0240)
Junior high school		–0.0938^***^	–0.1276^***^	–0.1342^***^
		(0.0242)	(0.0242)	(0.0242)
Senior high school		–0.1140^***^	–0.1657^***^	–0.1726^***^
		(0.0275)	(0.0276)	(0.0276)
College		–0.0233	–0.0935^***^	–0.1120^***^
		(0.0311)	(0.0313)	(0.0313)
Graduate		–0.1837^***^	–0.2791^***^	–0.2965^***^
		(0.0692)	(0.0681)	(0.0679)
Political identity		0.1889^***^	0.1438^***^	0.1346^***^
		(0.0217)	(0.0217)	(0.0217)
Household registration		–0.1424^***^	–0.1327^***^	–0.1166^***^
		(0.0139)	(0.0139)	(0.0140)
Family economic level			0.1638^***^	0.1596^***^
			(0.0073)	(0.0073)
Medical insurance				0.2816^***^
				(0.0210)
Province	YES	YES	YES	YES
Year	YES	YES	YES	YES
N	28,239	28,239	28,239	28,239

Robust standard errors are in parentheses; ^*^, ^**^ and ^***^ denote significance at the 10%, 5% and 1% level, respectively. Data are from the Chinese Social Survey [51] for 2013, 2015 and 2017.

**Table 4:** Attitudes toward Internet and medical service satisfaction.

Variable	Dependent Variable: MSS	
	(1)	(2)
Attitudes toward Internet information	0.0490^***^	
	(0.0182)	
Attitudes toward netizens		0.0460^***^
		(0.0175)
Control variable	YES	YES
Province	YES	YES
Year		
N	6466	6579

Robust standard errors are in parentheses; ^***^ denotes significance at the 1% level. Data are from the Chinese Social Survey [51] for 2013, 2015 and 2017.

**Table 5:** The effect of Internet use people’s different aspects of medical service evaluation.

Variable	Dependent Variable						
	X1	X2	X3	X4	X5	X6	X7
	(1)	(2)	(3)	(4)	(5)	(6)	(7)
IU	–0.1107^***^	–0.0822^**^	–0.1111^***^	–0.0679^*^	0.0118	–0.1034^***^	–0.0239
	(0.0359)	(0.0367)	(0.0370)	(0.0368)	(0.0361)	(0.0370)	(0.0390)
Control variable	YES	YES	YES	YES	YES	YES	YES
Province	YES	YES	YES	YES	YES	YES	YES
Year	YES	YES	YES	YES	YES	YES	YES
N	7560	7552	7557	7553	7533	7559	7553
Variable	X8	X9	X10	X11	X12	X13	
	(8)	(9)	(10)	(11)	(12)	(13)	
IU	–0.1616^***^	–0.2000^***^	–0.1552^***^	–0.1766^***^	–0.2064^***^	–0.1455^***^	
	(0.0397)	(0.0388)	(0.0374)	(0.0373)	(0.0195)	(0.0248)	
Control variable	YES	YES	YES	YES	YES	YES	
Province	YES	YES	YES	YES	YES	YES	
Year	YES	YES	YES	YES	YES	YES	
N	7412	7509	7433	8809	27,595	17,931	

Robust standard errors are in parentheses; ^*^, ^**^ and ^***^ denote significance at the 10%, 5% and 1% level, respectively. Data are from the Chinese Social Survey [51] for 2013, 2015 and 2017.

**Table 6:** Internet use and medical service satisfaction: Subdivided sample analysis (by gender, urban-rural and year).

Variable	Dependent Variable: MSS						
	Male	Female	Urban	Rural	2013	2015	2017
	(1)	(2)	(3)	(4)	(5)	(6)	(7)
IU	–0.1665^***^	–0.1066^***^	–0.1277^***^	–0.1240^***^	–0.1348^***^	–0.0993^***^	–0.1331^***^
	(0.0255)	(0.0238)	(0.0237)	(0.0263)	(0.0323)	(0.0305)	(0.0280)
Control variable	YES	YES	YES	YES	YES	YES	YES
Province	YES	YES	YES	YES	YES	YES	YES
Year	YES	YES	YES	YES			
N	12,794	15,445	13,461	14,778	9161	9390	9688

Robust standard errors are in parentheses; ^***^ denotes significance at the 1% level. Data are from the Chinese Social Survey [51] for 2013, 2015 and 2017.

**Table 7:** Internet use and medical service satisfaction: Subdivided sample analysis (by education).

Variable	Dependent Variable: MSS		
	Primary School and Below	Junior and Senior High School	Undergraduate and above
	(1)	(2)	(3)
IU	–0.1636^***^	–0.1382^***^	–0.1053
	(0.0428)	(0.0204)	(0.0650)
Control variable	YES	YES	YES
Province	YES	YES	YES
Year	YES	YES	YES
N	10,173	14,031	4035

Robust standard errors are in parentheses; ^***^ denotes significance at the 1% level. Data are from the Chinese Social Survey [51] for 2013, 2015 and 2017.

**Table 8:** Internet use and medical service satisfaction: Ordered logit and probit estimation.

Variable	Dependent variable			
	MSS		MSS1	
	Ordered Logit		Probit	
	(1)	(2)	(3)	(4)
IU	–0.2437^***^	–0.2289^***^	–0.0817^***^	–0.1276^***^
	(0.0214)	(0.0296)	(0.0162)	(0.0221)
Control variable	NO	YES	NO	YES
Province	YES	YES	YES	YES
Year	YES	YES	YES	YES
N	28,239	28,239	28,239	28,239

Robust standard errors are in parentheses; ^***^ denotes significance at the 1% level. Data are from the Chinese Social Survey [51] for 2013, 2015 and 2017.

**Table 9:** Internet use and medical service satisfaction (propensity score matching).

Matching Methods	2-nearest Neighbor Matching	Radius Matching	Kernel Matching	Local linear Regression Matching
ATT	–0.3389^***^	–0.3661^***^	–0.3332^***^	–0.3772^***^
	(0.0867)	(0.0729)	(0.0697)	(0.0860)
Control variables	YES	YES	YES	YES
Sample number of treatment group	10,427	10,427	10,427	10,427
Sample number of Control group	17,812	17,812	17,812	17,812

Standard errors are in parentheses; ^***^ denotes significance at the 1% level. Data are from the Chinese Social Survey [51] for 2013, 2015 and 2017.

**Table 10:** Internet use and medical service satisfaction (placebo test).

Variable	Dependent Variable: MSS			
	(1)	(2)	(3)	(4)
Watching TV	0.0247^***^			
	(0.0095)			
Listening radio		0.0124		
		(0.0082)		
Reading newspapers			0.0028	
			(0.0076)	
Reading books				0.0160^**^
				(0.0080)
Control variable	YES	YES	YES	YES
Province	YES	YES	YES	YES
Year	YES	YES	YES	YES
N	9160	9152	9152	9156

Robust standard errors are in parentheses; ^**^ and ^***^ denote significance at the 5% and 1% level, respectively. Data are from the Chinese Social Survey [51] for 2013 (because only the Chinese Social Survey in 2013 included information about the respondents’ use of traditional media).
